# Microbiome yarns: microbiomology of curly and straight hair

**DOI:** 10.1111/1751-7915.12694

**Published:** 2017-02-27

**Authors:** Kenneth Timmis, Franziska Jebok, Fabio Rigat, Willem M. de Vos, James Kenneth Timmis

**Affiliations:** ^1^ Institute of MicrobiologyTechnical University BraunschweigBraunschweigGermany; ^2^ Institute for Educational ScienceUniversity of FreiburgFreiburgGermany; ^3^ Department of StatisticsUniversity of CoventryCoventryUK; ^4^ Laboratory of MicrobiologyWageningen UniversityWageningenThe Netherlands; ^5^ Department of Bacteriology and ImmunologyUniversity of HelsinkiHelsinkiFinland; ^6^ MSc Health Policy StudentDepartment of Surgery and CancerImperial CollegeLondonUK

## Part 1

*BBZ, Studio 7A, BBZ Plaza, Burbank, 7.30 pm: Abigail Repor‐Tastory, Discovery Presenter, turns to face the camera*: Good evening and welcome to a new episode of “Discoveries that Change our Lives”. Our guest this evening is Dr. Anastasia Noitall‐Most^1^ from the Streber Elite University of Los Angeles. Good evening Dr. Noitall‐Most *(shaking hands)* and thank you for appearing on the program.

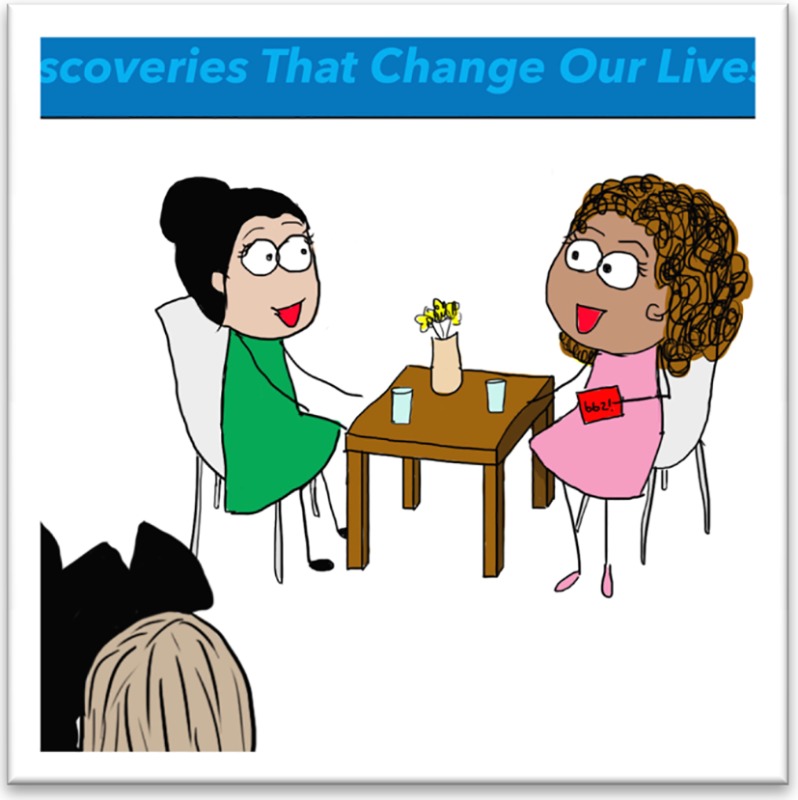

*Dr. Noitall‐Most:* Good evening Abi; it is always a pleasure to be here.

*Ms. Repor‐Tastory:* Ani – a seemingly sensational breakthrough that will allow us to choose whether we sport curly or straight hair was recently reported in the respected journal Natural Science – is it credible?

*Dr. Noitall‐Most:* Well, as you know, the previous theory about hair type was that it is determined by the shape of the hair follicle – if you like, the hair production centre. Straight follicles were thought to produce straight hair, whereas curved follicles thought to produce hooked hair shafts that give curly hair. However, the follicle shape theory was based purely on correlation and the new results have consigned it to the bin.

*Ms. Repor‐Tastory:* Gosh! So this discovery is really important. Ani: how was it made?

*Dr. Noitall‐Most:* Well Abi, it is, as most breakthroughs are, the result of a bright individual (in this case, a researcher in a global personal care products company – we'll not mention the name, to avoid any impression of endorsing it) connecting apparently unrelated threads of knowledge. The first is that, for reasons previously unknown, hair curling tendency, known in the business as HCT, can change dramatically with age, medication, etc. The second is a study of the microbiome of the scalp‐hair follicle microenvironment which revealed that certain types of bacteria, called enterobacteria (EB), are present in different amounts in people with curly hair and with straight hair. This suggested that EB may play a role in determining HCT.

*Ms. Repor‐Tastory:* Yes, Ani, but we have heard so much recently about people drawing all manner of silly conclusions from correlations, and from a slew of inane and/or poorly‐designed trials. How is this study different?

*Dr. Noitall‐Most:* Abi, you are absolutely right! However, in this case, the science is quite strong. It turns out that the EB identified have been shown to produce *curli fimbriae* 2 – these are surface projections on the bacteria, a bit like hair on our bodies, that have the property of causing hair to curl. A subsequent large survey confirmed that people with curly hair have a higher count of EBs around their hair follicles.

*Ms. Repor‐Tastory:* But this is still only a correlation and….

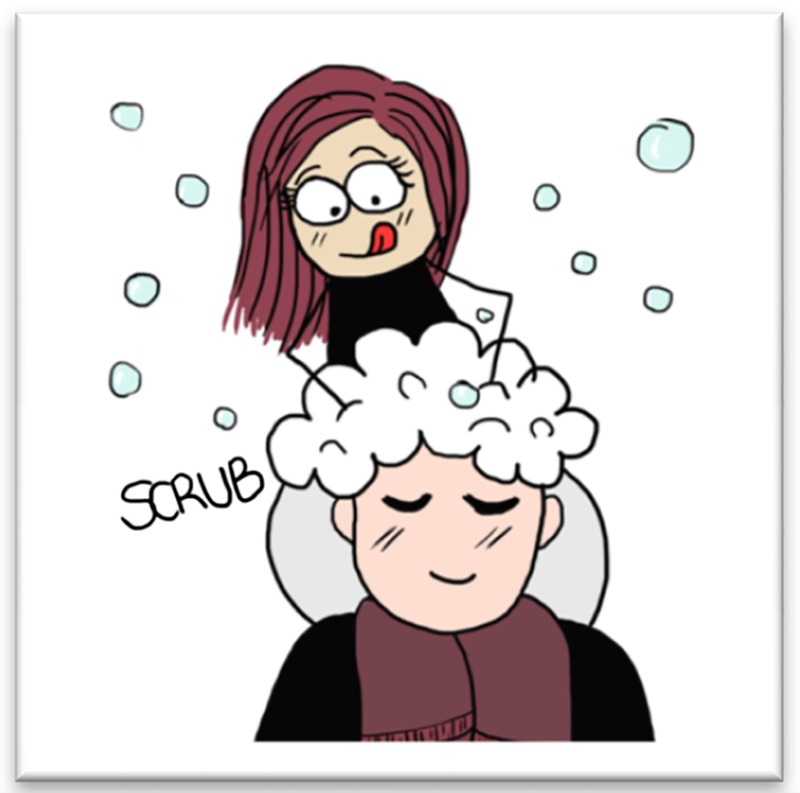

*Dr. Noitall‐Most, breaking in and in a slightly irritated tone:* Indeed, but, as I was about to say, the people from F'Real Hair Care^3^ (ooopss: unintended!) then did the clincher: they took hair microbiome samples from unwashed heads of a curly‐haired group, and the same from a straight‐haired group, gave all group members a hair wash to remove some of the hair microbiome, then immediately afterwards rubbed the samples – so‐called *hair microbiome transplants* – into the scalps of the donors in a double blind, randomised trial, and monitored any changes in hair type occurring over a 6‐week period. The amazing result was that, in 73% of the cases, straight hair turned curly if it had received a “curly transplant”, and curly hair turned straight if it had received a “straight transplant”! Even more amazing was that a quantitative microbiome analysis after 6‐weeks showed that the straight‐curly characteristic, HCT, correlated almost perfectly with the level of EBs in the follicle areas, including in those outliers for which the transplant did not work!

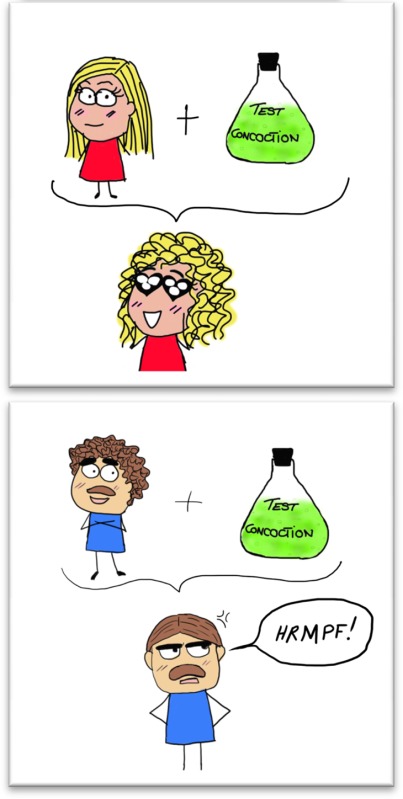

*Ms. Repor‐Tastory:* But didn't I read somewhere of an adverse effect of the transplant?

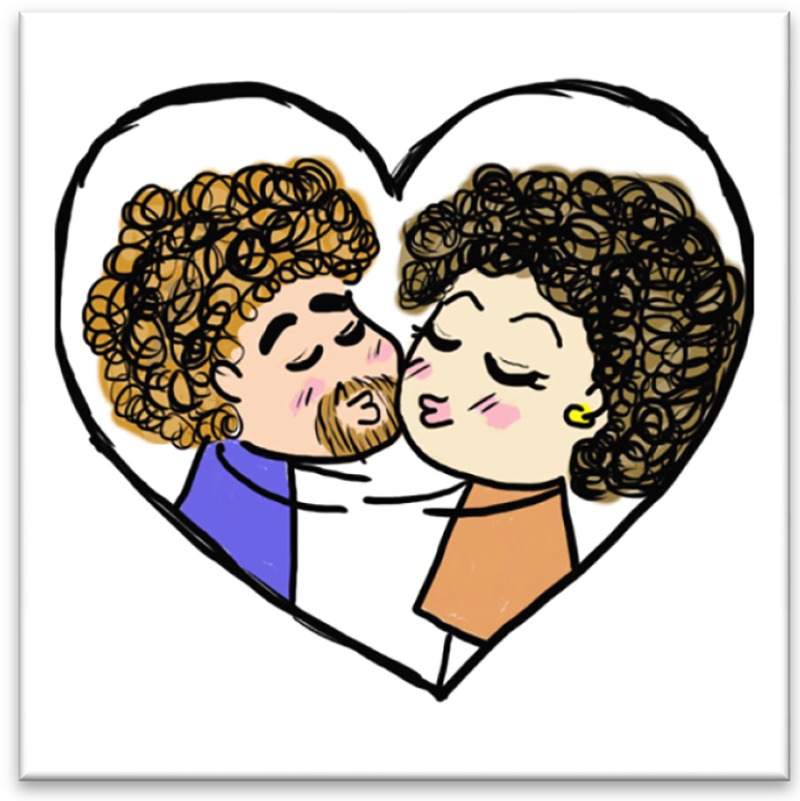

*Dr. Noitall‐Most:* Correct! Apparently, during the dose‐response trial, two members, who unknowingly had received the highest doses of the curly transplant, became…..let's say amorously involved. And during a somewhat intimate tête‐à‐tête, their super curly heads of hair became impossibly tangled together – the trial chief called it a *Velcro situation* – such that they became glued together at their heads! It was a tad embarrassing for them, firstly in having to seek help under rather compromising circumstances, to get separated by having the joined locks cut off, and subsequently in explaining their change in appearance to their regular partners. This part of the trial was immediately aborted, of course, but the trial itself was successful in demonstrating that lower, non‐problematic doses are effective in changing HCT.

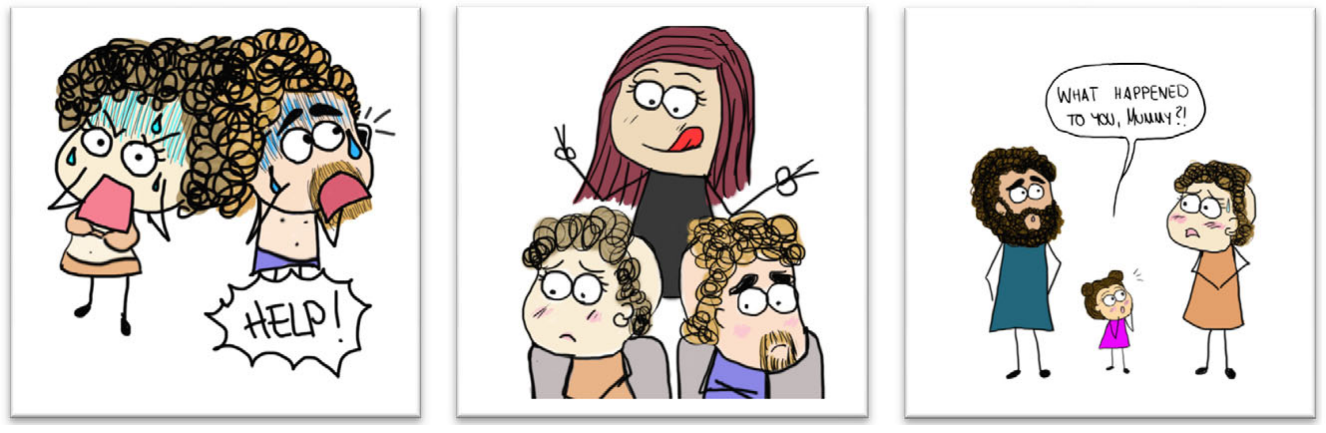

*Ms. Repor‐Tastory, giggling:* Wow! So when can I get my “straight transplant”?

*Dr. Noitall‐Most:* According to a press release made last week by F'Real Hair Care (oh, sorry!), the first products will be marketed within one year in hairdressing salons, to be applied in a scalp massage product, after hair bleaching‐colouring. The reason for this is that the company found that the success rate rises from 73% to 91% if the transplant is made after a major microbiome perturbation, such as occurs during the hair bleaching process, which gives the transplant a better chance of taking. I assume they will carefully monitor and gain experience from this restricted application, before making it generally available for home use, probably in the form of a gel product.

*Ms. Repor‐Tastory:* Fantastic! On that note, we'll take a break and return to this fascinating story in a few minutes.

++++++++++++

## Part 2

*Ms. Repor‐Tastory:* Welcome back viewers! Now, Ani, could you please explain to our readers how this curli stuff works?

*Dr. Noitall‐Most:* Yes, Abi. In fact, curli proteins have a multitude of properties,^2^ including sticking to many types of surfaces. Some curli‐producing EBs cause infections, like diarrhoea or urinary tract infections…

*Ms. Repor‐Tastory, promptly crossing her legs:* Ooohh!

*Dr. Noitall‐Most:* …and curli plays an essential role in the infection process, in both humans and plants.^2^ Interestingly, the sticking property reflects the fact that they aggregate together, which makes them amyloid proteins of the type causing Alzheimer's.

*Ms. Repor‐Tastory, scratching her head:* Oh, my!

*Dr. Noitall‐Most:* …But no worries, Abi: most EBs, including those found in the scalp microbiome, are generally harmless. So how does curli affect hair curling? As I mentioned earlier, EBs on the scalp release curli protein which, in the presence of hair shafts, forms fibres that curl around the hair – we think by binding to keratin, a protein found in skin, nails, hair and horns – the so‐called *horny protein*. When it does this, it bends the hair shaft. When a lot of it is produced by a lot of EBs, a lot of bending is done, and hey presto – curls are formed!

*Ms. Repor‐Tastory, smiling:* Now that is remarkable! So curli and horny marry and give birth to wavy? Amazing! I am sure our viewers are thinking: well – our body hair tends to be curly whatever the form of the hair on our heads. Is that anything to do with EBs and curli?

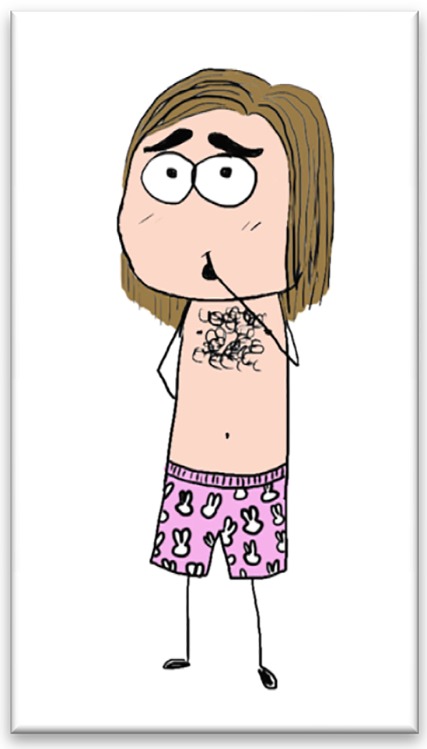

*Dr. Noitall‐Most:* Absolutely, Abi: excellent point! As one might predict, EB numbers in axial regions and, in the case of males, on other body surfaces, are systematically high. In fact, the renowned mathematician Professor Fidget Jones has derived a very simple formula^4^ that expresses the relationship between HCT and the fraction of EB in the hair microbiome, and that takes into account key environmental parameters.

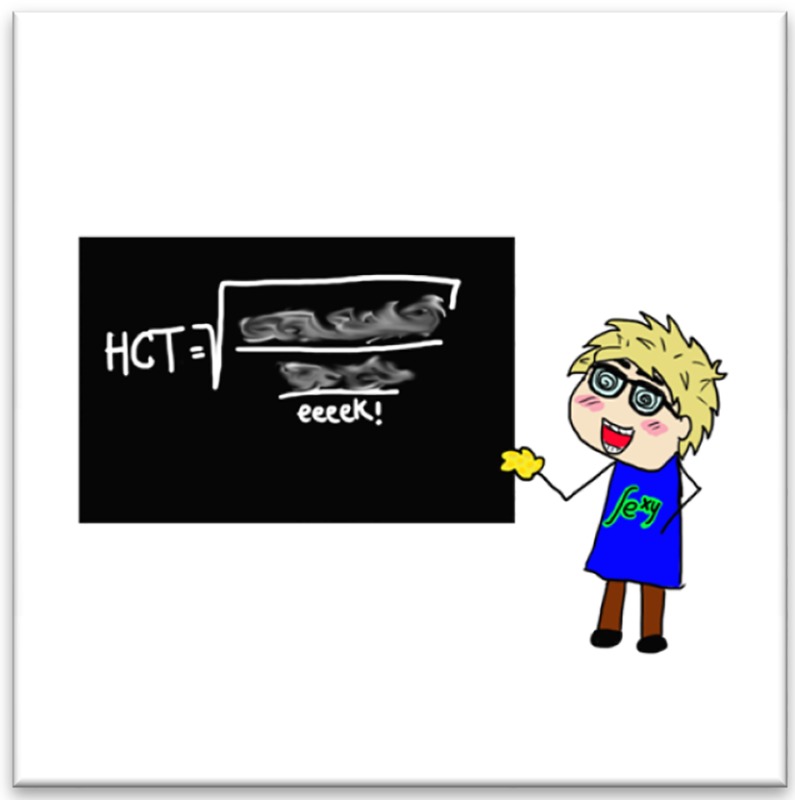

*Ms. Repor‐Tastory:* So if I double the number of EBs on my scalp, I'll double the curliness of my hair?

*Dr. Noitall‐Most:* No, not exactly. One thing that researchers found out is that there is not a one‐to‐one relationship between EB numbers and degree of curliness. Apparently, production of the curli protein is influenced by something called “quorum sensing” which, stated simply, means that the more EBs on the hair shaft, the more curli protein each one produces. It is typical group behaviour, for example, of a football stadium crowd, in which a few potential bottle throwers tend to save their energies for wife‐beating at home, whereas many together will cause a riot on the pitch.

*Ms. Repor‐Tastory:* Oh, ok: so EBs are a bit like football rowdies with curli acting as their missiles?

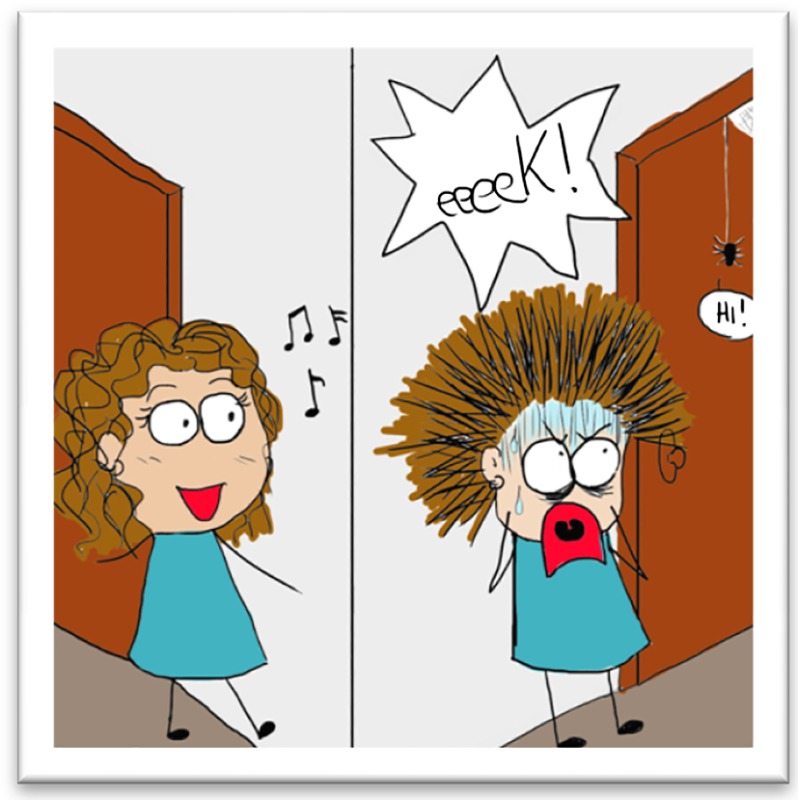

*Dr. Noitall‐Most:* Well: that is a bit of over‐dramatisation. Of course, the equation does not take into account isolated events, like frights, which can stiffen hair and reduce the curl factor, or hair treatments, like the application of gels, etc. Viewers can get more in‐depth information about these aspects from my website, the address of which will be shown in the credits at the end of the program^5^.

*Ms. Repor‐Tastory:* What a fascinating story! We'll take another break and return in a few moments to the final part of this incredible story.

++++++++++++++++++

## Part 3

*Ms. Repor‐Tastory:* Welcome back viewers! In this final part of the curly hair story, we want to learn from Dr. Noitall‐Most about the business of scalp microbiome transplants and what the future holds. Ani: would you please tell us what we can expect in the future?

*Dr. Noitall‐Most:* With pleasure! The first item to mention is that, although EBs are natural products, and hence not controversial as additives in, say, food or personal care products, there is an expectation that all types of microbiome transplant will eventually fall under regulatory scrutiny and safety regulation in the not‐too‐distant future. For this reason, R&D is currently directed at providing a single, extremely well characterised EB that can substitute for whole microbiome transplants, and whose safety and efficacy is incontestably documented. The potential problem is an ecological one: will a single strain of EB be ecologically competitive in all scalp environments, and thereby do its curly work? The other side of this coin is that it will be necessary to find a single microbiome member effective at displacing curli‐producing EBs from curly hair, the so‐called *disEB*, and at creating straight hair.

*Ms. Repor‐Tastory, straight‐faced:* I see. So not all is straight‐forward (sorry!) in the curly business?

*Dr. Noitall‐Most:* No…quite. However, many countries do not have comprehensive regulatory systems in place, so, from the business point of view, there is not much hindrance to product marketing from a global perspective.

*Ms. Repor‐Tastory:* I see. So where are currently the most promising markets?

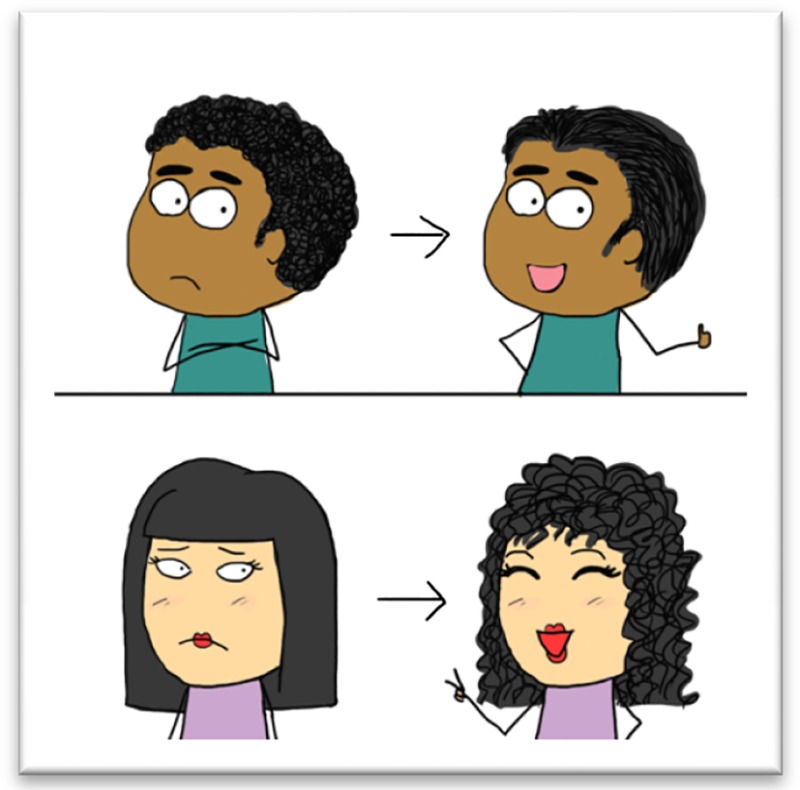

*Dr. Noitall‐Most, smiling:* Oh: I'd have thought this was obvious. Much of the world is seriously curly‐haired and the women in particular spend an enormous amount of time and money straightening their hair. And much of the remainder is straight‐haired and desirous of curly locks. In fact, as you will be aware, it is a normal human trait to be dissatisfied with the looks our parents pass on to us, and to want to change them. Given that the global population is currently around 7.5 billion, even considering only the 20‐50 age group in 20% of the population, there is a considerable market for hair microbiome treatments.

*Ms. Repor‐Tastory:* Wow: this is mind‐blowing! What business potential!

*Dr. Noitall‐Most, grinning:* It would seem so…and we should also keep in mind that any hair treatment, including repeated washing, but especially visits to the hair dresser, is likely to reduce over time the numbers of EB or *disEB*, so repeat, probably life‐long, treatments will be the norm, which will, of course, be good for the business.

*Ms. Repor‐Tastory:* What a perfect situation!

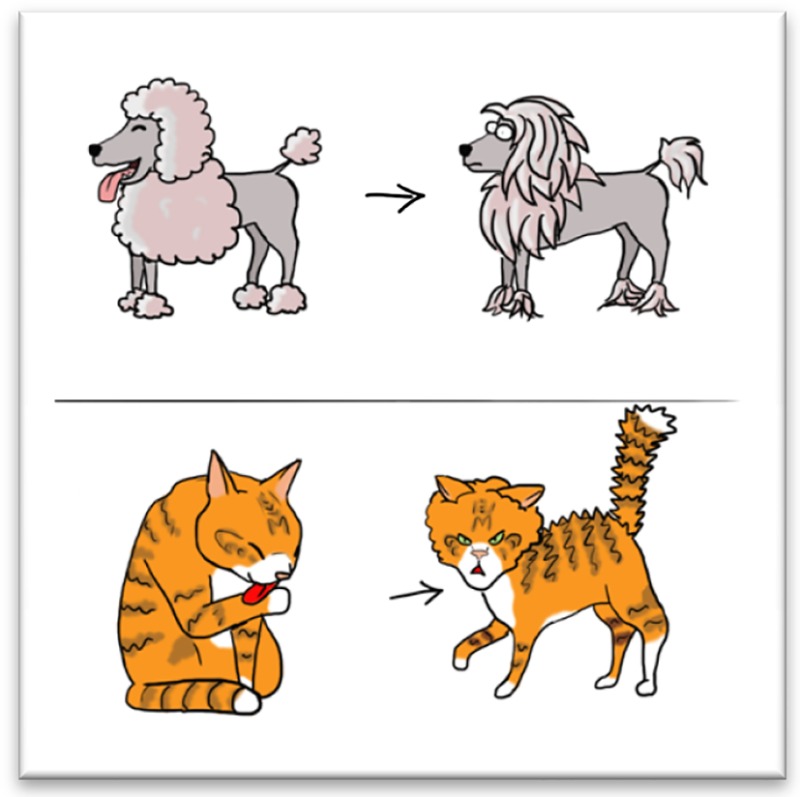

*Dr. Noitall‐Most, grinning widely:* Yes, but that is not the end of the story. A major pet grooming products company has now started marketing dog and cat versions of the curly and straight transplants, and is aggressively promoting new pet fashion trends to create a market. I suspect we will start to see some rather unusual household pet styles in the near future. By the way, the HCT formula developed by Fidget Jones requires additional terms when applied to pets, to take into account the tendency to curl up in front of the fire, which reduces the production of curli, as indeed do the continual self‐grooming by licking, exhibited by cats, and the rolling in any available smelly dirt, exhibited by dogs.

*Ms. Repor‐Tastory:* Goodness! Are there any flies in the ointment?

*Dr. Noitall‐Most, gravely:* Well, as I mentioned, there is an issue of temperature: curli protein tends to be produced better at temperatures below 30°C by most strains of EB, which is fine for folks living in Arctic/sub‐Arctic climes, but not for the majority of large populations with either curly or straight hair. However, some strains, unfortunately mostly pathogenic strains, produce curli at human body temperature, so there is a search right now for a non‐pathogenic strain producing curli at body temperature that would not raise the hackles of regulatory agencies.

*Ms. Repor‐Tastory, composing herself*: errrm…are there any known safety issues that might negatively impact the business model?

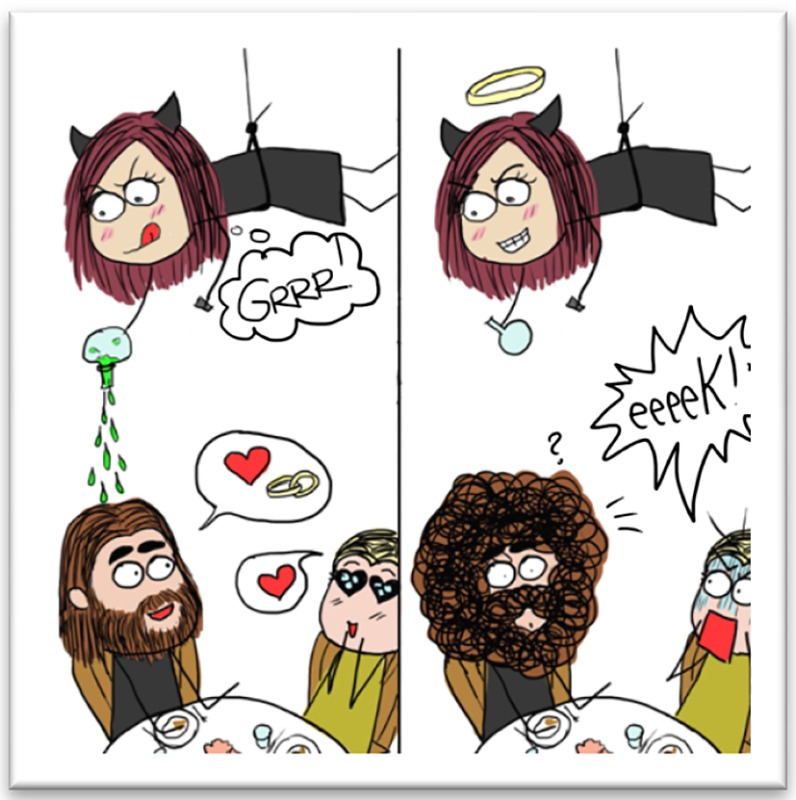

*Dr. Noitall‐Most:* At present, there are no known significant safety issues of either non‐pathogenic EBs or curli, apart from potential exacerbation of inflammatory conditions of the skin, like dermatitis, psoriasis, etc., because curli has been shown to have inflammation‐inducing capacity. From medical and ecological theoretical points of view, there are no obvious risks, though only usage over a number of years will tell. Of course, as with any new development, there exists a possibility of misuse, such as accidental or deliberate overdosing, either self‐inflicted, or perpetrated by a rival, jealous ex‐partner, or even drunken friends, to convert straight elegance to curly mop (and potentially of course a *Velcro situation*).

*Ms. Repor‐Tastory:* Well: on that cautionary but responsible note, we bring to an end this fascinating topic of hair microbiome transplants. Thank you so much Dr. Noitall‐Most for being with us tonight and we look forward to seeing you again on this programme to enlighten us on another topic in the series of “Discoveries that Change our Lives”.

*Ms. Repor‐Tastory, off camera, off microphone:* Thanks a million, Ani! Sorry – must rush: need to contact my broker before F'Real shares go through the roof!!

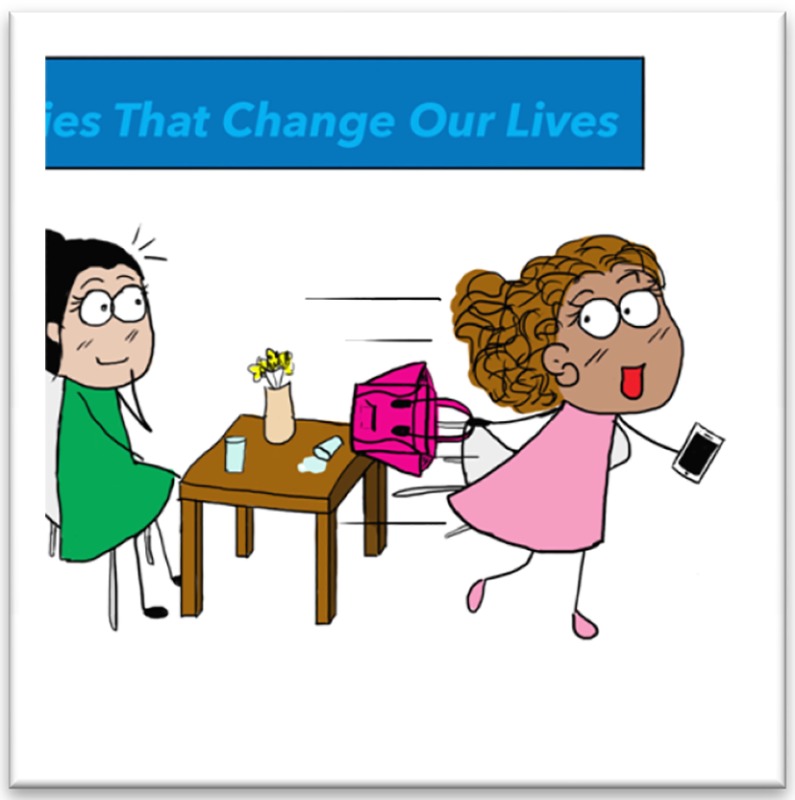

## Conflict of interest

None declared.

## Explanatory notes for online version at www.theabsurdmicrobe.com

1
*Health Warning*. This article is a figment of the authors’ imagination; any serious contemplation may perturb the reader's mental harmony.

2 Any possible resemblance to individuals or organisations, real or fictional, is entirely coincidental and unintended.

3 dedicated to the brilliant pioneers of curli research, especially Staffan Normark, whose originality and insightfulness have revealed to us the properties and functional glory of this remarkable hairy protein, to the intrepid leaders of microbiome research, and to the memory of Dr. Noitall, figment of the imagination of the late Dan Koshland (e.g. Koshland, D. E., Jr. 1994. Editorial: A simple proposal for health care rationing. Science **264**: 1515)

4 youngest daughter of the renowned Dr. Noitall.

5
www.noitallmost.com

